# Complete chloroplast genome sequences of six lines of *Echinochloa colona* (L.) link

**DOI:** 10.1080/23802359.2016.1261612

**Published:** 2017-01-09

**Authors:** James P. Hereward, Jeff A. Werth, David F. Thornby, Michelle Keenan, Bhagirath Singh Chauhan, Gimme H. Walter

**Affiliations:** a School of Biological Sciences, The University of Queensland, Brisbane, Queensland, Australia;; b Queensland Department of Agriculture and Fisheries, Leslie Research Centre, Toowoomba, Queensland, Australia;; c Innokas Intellectual Services, Coomera, Queensland, Australia;; d The Centre for Plant Science. Queensland Alliance for Agriculture and Food Innovation (QAAFI), The University of Queensland, Gatton, Queensland, Australia

**Keywords:** *Echinochloa*, millet, glyphosate resistance, weed

## Abstract

Barnyard grass (*Echinochloa colona*, (L.) Link) is the wild relative of barnyard millet (*E. frumentacea* (Roxb.) Link). This species, widely distributed globally, is an agricultural weed and has developed resistance to several herbicides including glyphosate. This paper presents the complete chloroplast sequences of two haplotypes (139,718 bp & 139,719 bp) sequenced from six lines of *E. colona* from Australia. The *E. colona* chloroplast sequence is very similar to that of *E. frumentacea* (163–169bp =0.12% differences across the genome). The gene content, arrangement, and the inverted repeat structure is the same as in the other species of *Echinochloa* sequenced to date.

Barnyard grass (*Echinochloa colona* (L.) Link) is considered to be the wild ancestor of barnyard millet (*E. frumentacea* (Roxb.) Link), also known as Indian or sawa millet. The taxonomic status of these two hexaploid species is still under discussion, and hybrids show varying degrees of sterility (Yabuno 1962). In Australia, glyphosate-resistant populations of *E. colona* were first detected in 2007 and currently over 100 resistant populations have been confirmed (Cook *et al*. 2008, Heap 2016, Preston 2016).

In this study, six different lines of *E. colona* were sequenced as part of an ongoing investigation into glyphosate-resistance mechanisms, and here we report the complete chloroplast genomes for these lines. The seed that gave rise to line PLG3 was collected in 2011 near Boggabilla (New South Wales, Australia), PJ55 near North Star (New South Wales, Australia), TC near Tamworth (New South Wales, Australia), QBG4 near Karara (Queensland, Australia), QBG1 near Dalby (Queensland, Australia), and QBG3 near Dulacca (Queensland, Australia). These lines had varying levels of glyphosate resistance and the exact locations have been withheld to protect landholders. DNA was extracted from leaf material of two individuals grown from each line; a representative voucher of line QBG4 is held at The University of Queensland and is available from the corresponding author. Genomic sequencing libraries were constructed using the NebNext Ultra DNA kit (New England Biolabs, MA) and PE125 Illumina sequencing was performed by Novogene (Beijing, China). The chloroplast sequences were assembled in Geneious v9.1.3 (http://www.geneious.com, Kearse et al. 2012) by mapping reads to the complete *Echinochloa crus-galli* chloroplast sequence (KJ000047, Ye et al. 2014), followed by iterative re-mapping to the consensus. The final consensus sequences were checked manually to ensure correct mapping distances across the assembly. All available complete chloroplast sequences for the genus *Echinochloa* were downloaded from Genbank and aligned with the new sequences using MAFFT (Katoh & Standley 2013). The GTR + I + G model of nucleotide substitution was found to be the most likely by jmodeltest2 (Darriba et al. 2012). A Bayesian phylogenetic tree was produced using MrBayes (Huelsenbeck & Ronquist 2001) under this substitution model with *Amphicarpum muhlenbergianum* used as an outgroup (Figure 1).

**Figure 1.**
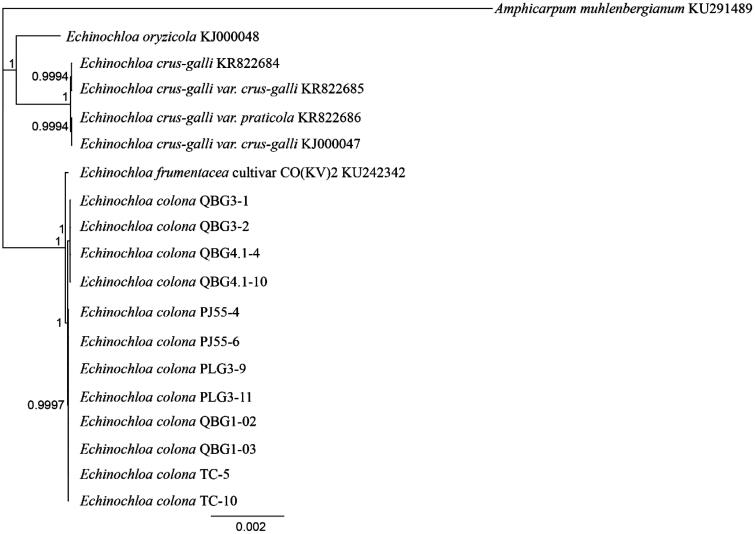
Phylogenetic tree produced using the Bayesian estimation (Mr. Bayes) of all complete chloroplast genomes from the genus *Echinochloa* using *Amphicarpum muhlenbergianum* as an outgroup, node labels indicate the posterior probability after 1 × 10^6^ iterations.

Two distinct haplotypes were present within the six lines sequenced in this study, all individuals from lines QBG3 and QBG4 shared one haplotype (KX870854, length =139,719bp), with all other individuals having the second haplotype (KX870853, 139,718bp, Figure 1). These two haplotypes differed by only 11 single-nucleotide polymorphisms (SNP’s) and one indel. The chloroplast genome sequence of *E. colona* is very similar to that of *E. frumentacea* (KU242342, Perumal et al. 2016), with *E. frumentacea* having two deletions (20bp and 112bp, in non-coding regions) relative to *E. colona*, and a mean of 34 single site differences (indels and snps) to the six *E. colona* lines. No differences in gene content or arrangement were detected across all the four species from this genus that now have a complete chloroplast sequence available.
